# Restricted mobility of Dnmt1 in preimplantation embryos: implications for epigenetic reprogramming

**DOI:** 10.1186/1471-213X-5-18

**Published:** 2005-08-24

**Authors:** Maik Grohmann, Fabio Spada, Lothar Schermelleh, Natalia Alenina, Michael Bader, M Cristina Cardoso, Heinrich Leonhardt

**Affiliations:** 1 Department of Biology II, Ludwig Maximilians University Munich, Grosshadernerstr. 2, 82152 Planegg-Martinsried, Germany; 2 Max Delbruck Center for Molecular Medicine, Berlin, Germany; 3 Max-Planck-Institute for Molecular Genetics, Berlin, Germany

## Abstract

**Background:**

Mouse preimplantation development is characterized by both active and passive genomic demethylation. A short isoform of the prevalent maintenance DNA methyltransferase (Dnmt1S) is found in the cytoplasm of preimplantation embryos and transiently enters the nucleus only at the 8-cell stage.

**Results:**

Using GFP fusions we show that both the long and short isoforms of Dnmt1 localize to the nucleus of somatic cells and the cytoplasm of preimplantation embryos and that these subcellular localization properties are independent of phosphorylation. Importantly, photobleaching techniques and salt extraction revealed that Dnmt1S has a very restricted mobility in the cytoplasm, while it is highly mobile in the nucleus of preimplantation embryos.

**Conclusion:**

The restricted mobility of Dnmt1S limits its access to DNA and likely contributes to passive demethylation and epigenetic reprogramming during preimplantationdevelopment.

## Background

In mammals establishment and maintenance of DNA methylation patterns are crucial for embryonic development, cell differentiation, silencing of transposable elements, X inactivation and allele-specific expression of imprinted genes [1]. DNA methyltransferases (Dnmts) are responsible for establishment and maintenance of methylation patterns. In contrast to Dnmt3a and 3b, which catalyze *de novo *methylation of unmethylated DNA, Dnmt1 shows a preference for hemi-methylated DNA and is targeted to replication foci by binding to PCNA during S-phase [2-4]. Thus, Dnmt1 is thought to maintain genomic methylation through DNA replication by reproducing the cytosine methylation pattern of the parental DNA strand onto the newly synthesized strand.

Genomic methylation patterns undergo drastic changes during gametogenesis and early embryonic development. In the germ line, methylation patterns are erased early in development and gamete-specific ones are established during gametogenesis [5]. In the mouse zygote there is a drastic decrease of DNA methylation in the paternal genome within a few hours after fertilization (active demethylation) and both the maternal and paternal genomes undergo progressive demethylation during segmentation stages [6-9]. This is followed by establishment of new, tissue specific methylation patterns beginning around the time of implantation [9,10].

Different isoforms of Dnmt1 are encoded by the mouse *dnmt1 *locus. A longer isoform (Dnmt1L) is expressed in somatic and embryonic stem cells where it is strictly nuclear, except in post-mitotic neurons where it is also found in the cytoplasm [2,11,12]. A shorter, maternally contributed isoform lacking 118 amino acids at the N-terminus (Dnmt1S) is found in the cytoplasm of maturing oocytes and preimplantation embryos and enters the nucleus only transiently at the 8-cell stage [13-16]. The methylation maintenance function of Dnmt1 is shared by the long and short isoforms as the latter can rescue methylation patterns and differentiation potential in ES cells and mice lacking the former [11,17]. It is believed that retention of Dnmt1S in the cytoplasm of preimplantation embryos may prevent maintenance of gamete-specific methylation patterns, determining their erasure by passive demethylation and thus contributing to epigenetic reprogramming of the embryo. However, it is far from clear how methylation patterns at imprinted loci and transposable elements are maintained throughout preimplantation development and how Dnmt1S is prevented from entering the nucleus. Interestingly, during Xenopus early embryonic development a Dnmt1 isoform equivalent to the mouse long isoform is present in the nuclei and only limited demethylation occurs [18,19].

Here we investigated the localization of GFP fusions of the long and short Dnmt1 isoforms in mouse preimplantation embryos and directly compared their mobility in the nucleus and cytoplasm of living embryos.

## Results and discussion

**Figure 1:**
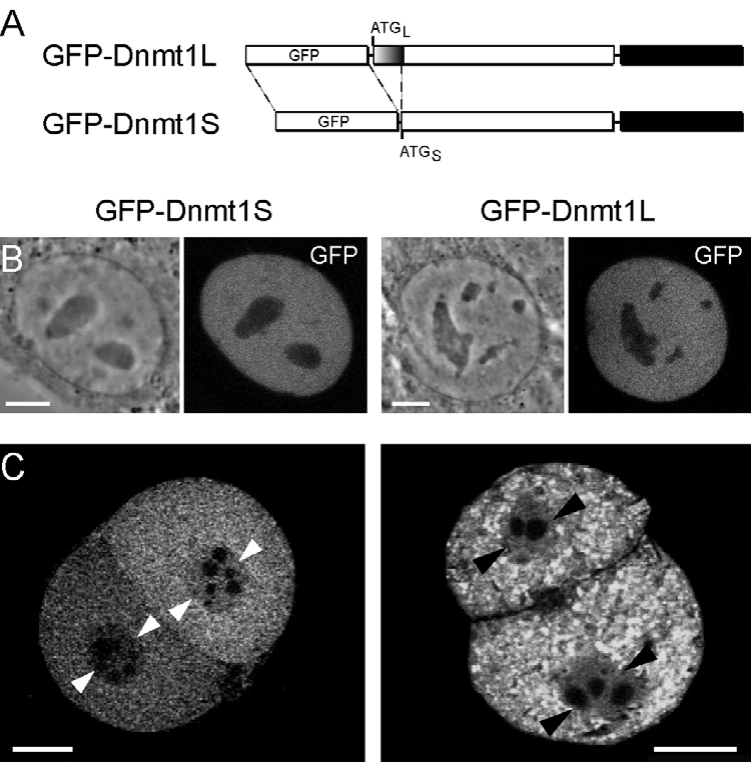
**Subcellular localisation of Dnmt1 isoforms in mouse somatic cells and preimplantation embryos**. A) Schematic representation of GFP-Dnmt1 fusion proteins. The start codons of the long (ATG_L_) and the short (ATG_S_) isoforms are indicated. The catalytic domain of Dnmt1 is in black. Subcellular localisation of GFP-Dnmt1 fusions in somatic cells (B) and 2-cell embryos (C). In B mouse C2C12 myoblasts were transfected with either the GFP-Dnmt1S (left pair of panels) or the GFP-Dnmt1L expression constructs (right pair of panels) and imaged by confocal microscopy. The left panel in each pair shows the phase contrast image, while the right panel shows GFP fluorescence (scale bars = 5 μm). In C the same expression constructs were microinjected in pronuclei at the 1-cell stage and embryos were further cultured until the 2-cell stage (scale bars = 20 μm). Both the short and the long isoforms of Dnmt1 are localised in the nucleus of myoblasts and in the cytoplasm of 2-cell embryos. Small amounts of fusion proteins in embryonic nuclei (arrowheads) are due to overexpression of Dnmt1 and consequent saturation of the cytoplasmic retention mechanism.

**Figure 3:**
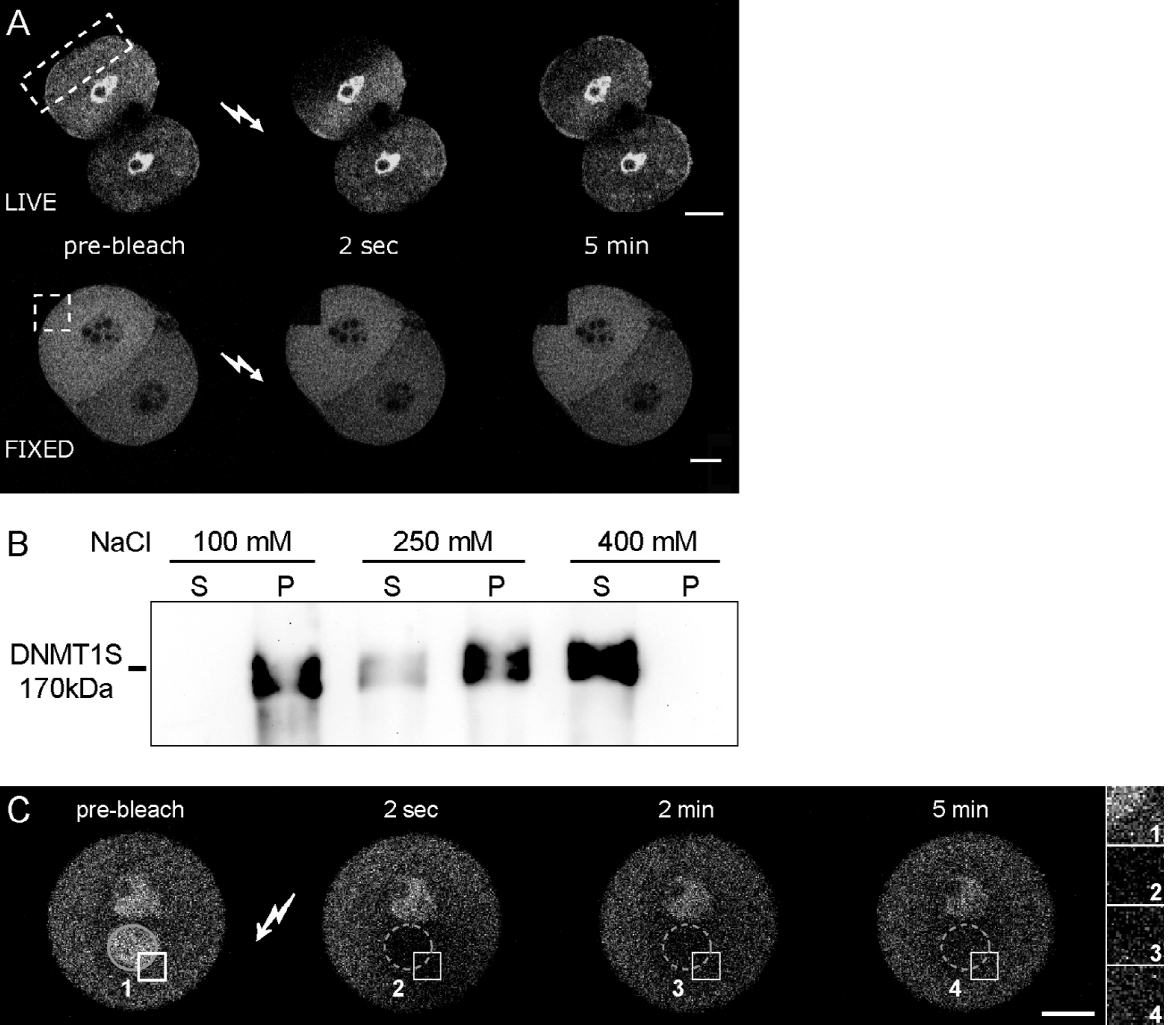
**Restricted mobility of GFP-Dnmt1S in the cytoplasm of mouse preimplantation embryos**. A) The GFP-Dnmt1S expression construct was microinjected in 1-cell stage embryos and a portion of the cytoplasm of one blastomere was bleached at the 2-cell stage. The upper row shows bleaching of a living embryo while the lower row shows a fixed control. Note that only regions immediately adjacent to the bleached area show decreased fluorescence. A very sharp bleaching boundary in fixed controls shows that such decrease is not due to poor sharpness of the bleaching beam, but to diffusion of GFP-Dnmt1S from adjacent sites. B) Salt extraction of endogenous Dnmt1S from 2-cell embryos. Soluble (S) and insoluble (P) fractions were analysed by immunoblotting with an anti-Dnmt1 antibody. C) Localisation dependent mobility of Dnmt1S in 1-cell embryos. A square bleached area (indicated) including a small fraction of the male pronucleus (outlined) was produced in a 1-cell embryo microinjected with the GFP-Dnmt1S construct. After bleaching no fluorescence remained in the entire pronucleus, while in the cytoplasm fluorescence was depleted only within and in proximity of the bleached area, indicating that the mobility of GFP-Dnmt1S is specifically restricted in the cytoplasm. Insets on the right show magnifications of the bleached area at the indicated time points. A) and C) show optical sections obtained by confocal microscopy (scale bars = 20 μm).

To directly compare the subcellular localization of the two Dnmt1 isoforms in cycling somatic cells and preimplantation embryos we expressed GFP-fusions of Dnmt1S and L (Fig. 1A and [4]) in both systems. After microinjection of the expression constructs in 1-cell embryos both fusion proteins were localized in the cytoplasm of preimplantation embryos (Fig. 1C), while they were exclusively nuclear in transfected mouse myoblasts (Fig. 1B). These results confirm earlier immunolocalization studies and indicate that the differential localization of the two Dnmt1 isoforms in somatic cells and embryos does not depend on the additional N-terminal 118 amino acids in Dnmt1L [13-16,20]. Nuclear localization of both isoforms in somatic cells is likely due to the fact that all active nuclear localization sequences are found within the region shared by the two Dnmt1 isoforms [15]. Indeed, overexpression of both isoforms by injection of 2–4 fold more plasmid DNA resulted in nuclear localisation of a fraction of the fusion proteins also in preimplantation embryos (Fig. 1C, 3A and 3C), suggesting a saturable cytoplasmic retention mechanism.

**Figure 2:**
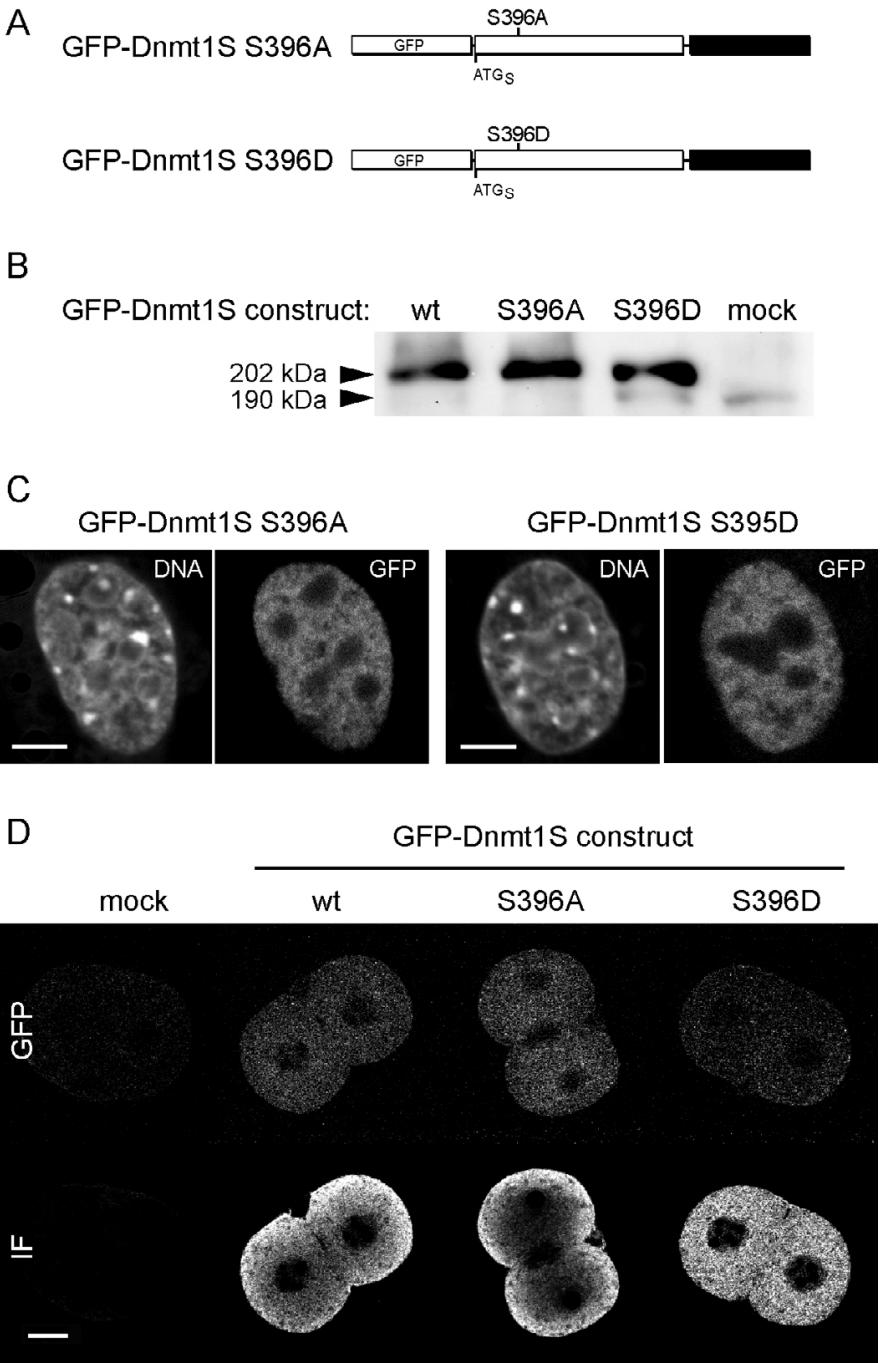
**Dnmt1S localisation is independent of phosphorylation**. A) Schematic representation of GFP-Dnmt1S phosphorylation mutants. B) Western blot of transfected Cos-7 cells probed with an antibody against the N-terminal domain of Dnmt1 [25] showing expression of GFP-Dnmt1s wild type (wt) and phosphorylation mutants S396A and S396D with the expected molecular mass. Weak bands at 190 kDa representing endogenous Dnmt1L are indicated. C) and D) localisation of GFP-Dnmt1S phosphorylation mutants in mouse somatic cells and preimplantation embryos, respectively. Both mutant proteins localise to the nucleus of somatic cells and to the cytoplasm of preimplantation embryos like wild type GFP-Dnmt1S (Fig. 1B and C). In D embryos were fixed and immunostained with an anti-GFP antibody detected with a red fluorescent secondary antibody. The upper row shows GFP fluorescence and the lower row shows the immunofluorescent signal (IF). The IF gradient from the periphery to the centre of some embryos is an optical artefact depending on signal intensity [15].

Posttranslational modification, in particular phosphorylation, is a well documented mechanism controlling the nuclear-cytoplasmic localization of a large number of proteins. The only posttranslational modification reported so far for Dnmt1 is phosphorylation of serine 514 (396 in Dnmt1S) [21]. To test whether the phosphorylation state of this residue determines the differential localization of GFP-Dnmt1S we generated mutations either mimicking the phosphorylated state (S396D) or preventing it (S396A; Fig. 2A). As shown in figure 2C and 2D neither mutation changed the nuclear localization of GFP-Dnmt1S in somatic cells or the cytoplasmic localization in preimplantation embryos.

To further investigate whether GFP-Dnmt1S is soluble or tightly bound to cytoplasmic structures in preimplantation embryos we tested fluorescence recovery after photobleaching of a defined region in the cytoplasm of microinjected embryos (Fig. 3A). Fluorescence recovery was very slow in living embryos with very little decrease of fluorescence in the non-bleached regions apart for those just next to the bleached area. This slow recovery could be explained by repeated association with fixed binding sites restricting its diffusion in the cytoplasm. We further assayed the stability of such binding of the endogenous Dnmt1S by salt extraction experiments (Fig. 3B). Essentially all Dnmt1S was found in the insoluble fraction with up to 250 mM NaCl, but was solubilized at 400 mM. This result provides biochemical evidence for strong interactions of Dnmt1S in early embryos. Since Dnmt1 is highly mobile in nuclei of somatic cells (data not shown) we directly compared the mobility of the short Dnmt1 isoform in the cytoplasm to that in the nucleus of the same embryo. For this we extensively bleached a region spanning across the nuclear-cytoplasmic boundary (Fig. 3C). Although only about one tenth of the nuclear volume was illuminated, the nuclear fluorescence was entirely depleted, indicating a high mobility of GFP-Dnmt1S molecules in the nucleus. In contrast, only regions within and close to the targeted area in the cytoplasm showed decreased fluorescence. As above, the rate of fluorescence recovery was slow, which is consistent with Dnmt1S binding to fixed structures in the cytoplasm. These results clearly show that Dnmt1S has a much higher mobility in the nucleus than in the cytoplasm of preimplantation embryos.

In summary, the subcellular localization of Dnmt1 does not depend on either the additional 118 amino acids at the N-terminus of Dnmt1L or the phosphorylation state at serine 396 of Dnmt1S. Previously, we showed that Dnmt1S fused to a SV40 NLS was still retained in the cytoplasm, ruling out that masking of the NLS of Dnmt1S could be the mechanism preventing nuclear localization like in the case of IκB preventing nuclear import of NF-κB. The biochemical extraction data indicate a strong binding of Dnmt1S, the overexpression experiments show that these binding sites are saturable and the fluorescence bleaching results demonstrate that GFP-Dnmt1S is specifically immobilized in the cytoplasm of preimplantation embryos.

## Conclusion

Taken together the data presented in this study argue for a strong binding of Dnmt1S to immobile structures in the cytoplasm of early embryos. Sequestration of Dnmt1 in the cytoplasm of embryos is likely to prevent full maintenance of methylation patterns during preimplantation development. Since mice expressing reduced levels of Dnmt1 protein already show severe genomic hypomethylation [22], even partial cytoplasmic retention of Dnmt1 could account for global passive demethylation and epigenetic reprogramming. At the same time, preferential targeting of remaining nuclear Dnmt1 could allow maintenance of parental methylation patterns at imprinted loci and silencing of endogenous retroviral elements.

## Methods

### DNA constructs

The expression construct for GFP-Dnmt1S (pEGMT1S) was derived by subcloning the cDNA insert from pEMT [23] into the Kpn I / Xma I site of the pEGFP-C1 vector (Clontech). The mutations S396D and S396A were introduced in pEMT by site directed mutagenesis using the QuikChange system (Stratagene) and the corresponding cDNA inserts were subcloned into the pEGFP-C1 vector as above. The expression construct for GFP-Dnmt1L (pEGMT1L) was obtained in two steps. First a 3.3 kb Xma I fragment from a PCR product spanning the whole N-terminal regulatory domain of Dnmt1L was cloned in the pEGFP-C1 vector to obtain pEGNMT. Next a Hind III / Psh AI fragment of pEGMT1S encompassing part of the EGFP coding sequence and the 5' part of the partial Dnmt1 cDNA till shortly downstream of ATG_4 _[11] was replaced with the corresponding fragment from pEGNMT, which contains the complete 5' sequence of Dnmt1L cDNA, including the part encoding the additional 118 amino acids. In all vectors transcription is under the control of the CMV promoter.

### Embryo collection, culture, microinjection and cell transfection

Mouse embryos were obtained from FVB/N superovulated females mated with FVB/N males. For superovulation females (3–4 weeks old) were injected intraperitoneally with 5 IU of pregnant mare serum (PMS) followed by intraperitoneal injection of 5 IU of human chorionic gonadotropin (hCG) 46–48 hours later. Matings were set up right after hCG injection. Zygotes were collected from the oviduct of 0.5 days post-coitum (d.p.c.) donors into M2 medium containing 300 μg/ml hyaluronidase to remove the cumulus cells. After washing in M2 medium the zygotes were transferred to M16 medium in microdrop cultures for further development up to the blastocyst stage in a humidified 5% CO_2 _incubator at 37°C. Culture conditions and media compositions were as described [24]. For injection, plasmid DNA was linearized, purified with standard glassmilk absorption techniques (Qiagen) and resuspended in microinjection buffer (8 mM Tris, pH 7.4 and 0.15 mM EDTA) at a final concentration of 10 μg/ml. DNA was injected into male pronuclei. C2C12 mouse myoblasts and Cos-7 cells were cultured and transfected as described [4,15].

### Immunofluorescence

Embryos were fixed with 3.7% formaldehyde in PBS for 10 min, permeabilised with 0.25% Triton X-100 in PBS and blocked with 0.2% fish skin gelatine in PBS for 30 min. Both primary and secondary antibodies were diluted in blocking solution and applied for 1 hr. The rabbit polyclonal antibody to GFP was from Abcam and was detected with an anti-rabbit secondary antibody conjugated with Alexa Fluor 568 (Molecular Probes). Embryos were washed with 0.1% NP-40 in PBS and mounted in Mowiol.

### Confocal Microscopy and Fluorescence Recovery after Photobleaching (FRAP)

Confocal images (2 μm thick optical slices) were taken with a confocal laser scanning microscope LSM510 (Zeiss) using a 488 nm and a 543 nm laser lines for GFP and Alexa Fluor 568 excitation and BP 500–530 nm and LP 570 nm filters for detection, respectively. For live cell microscopy, microinjected embryos were transferred to 8-well LabTek chambers (Nunc) containing M2 medium. FRAP experiments were performed at RT on a LSM510 using a 63 × Plan-Apochromat oil immersion objective (N.A. 1.4; Zeiss) with a 488 nm argon laser line. After acquisition of an initial (prebleach) optical section, a selected area was bleached for up to 30 s at 100 % laser power. Fluorescence recovery was then monitored by time lapse imaging 2 s and 5, 10 and 15 min after bleaching at low laser power.

### Salt extraction of mouse embryos

2-cell stage embryos were washed in PBS and transferred to a fresh Eppendorf tube in a 10 μl volume. 10 μl of 2 × EBC lysis buffer (100 mM Tris-HCl pH 8,0; 1% NP-40, pepstatin, aprotinin, leupeptin 2 μg/ml each, 200 μg/ml PMSF and either 200 mM, 500 mM or 800 mM NaCl) were added to each sample, followed by 5 min incubation on ice. 45 embryos were processed in each case. After centrifugation at 13,000 rpm and 4°C for 5 min soluble (supernatant) and insoluble (pellet) fractions were analysed by SDS-PAGE followed by immunoblotting and incubation with an antibody against the N-terminal domain of Dnmt1 [25].

## Abbreviations

GFP: green fluorescent protein; Dnmt: DNA methyltransferase.

## Authors' contributions

MG performed all the experimental work on mouse embryos. FS contributed fig. 2C and to writing of the manuscript. LS contributed fig. 1B. NA and MB provided crucial assistance to setting up experiments on mouse embryos. MCC and HL were involved in the conception and coordination of this project and the writing of the manuscript. All authors approved the final manuscript.
